# Sensitivity of low-frequency axial transmission acoustics to axially and azimuthally varying cortical thickness: A phantom-based study

**DOI:** 10.1371/journal.pone.0219360

**Published:** 2019-07-17

**Authors:** Florian Vogl, Mohit Patil, William R. Taylor

**Affiliations:** Institute for Biomechanics, Department of Health Sciences and Technology, ETH Zürich, Zürich, Switzerland; Shanghai Jiao Tong University Affiliated Sixth People's Hospital, CHINA

## Abstract

**Purpose:**

Cortical thickness (cTh) is one of the main factors determining a bone’s mechanical properties, and its quantification is therefore critical for understanding and monitoring bone pathologies such as osteoporosis. Axial quantitative acoustics (ax-QA) offers a non-radiative, non-invasive method to measure cTh. Even though previous works have ascertained ax-QA’s ability to measure azimuthally varying cTh, the effect of axially varying cTh remains unclear. Furthermore, previous experiments and theoretical predictions indicate that measurement of the fundamental flexural mode at low frequencies in the kHz range could increase sensitivity to cTh. However, due to the associated long wavelengths, the approximation of bone geometry as a tube could break down at such frequencies. The presented study therefore investigates a) the sensitivity of ax-QA measurements to cTh in the kHz-regime, b) the applicability of tube theory in this regime, and c) the effect of varying cTh along the long axis on the bone.

**Materials and methods:**

Axial-transmission acoustic measurements were performed at 3kHz on 14 bone phantoms with a femur-like cross-section and a) axially varying cortical thickness or b) axially and azimuthally varying cortical thickness (cTh-range: 1.5mm-7.5mm). Experimental results were compared to theoretical predictions based on an exact elastic tube theory.

**Results and discussion:**

Phase velocity measurements using low-frequency ax-QA exhibited a high sensitivity to local cTh less than 4mm, albeit with a complex, not yet understood pattern. Tube theory failed to predict the wave’s behavior in the kHz range, indicating that due to the corresponding long wavelengths the bone can no longer be approximated by a tube, thus requiring more faithful modelling of the bone geometry. The fact that results from both types of phantoms were similar (Pearson correlation coefficient: 0.94) further indicates that the slowly varying cTh along the bone’s long axis did not strongly affect wave propagation as measured by ax-QA measurements.

## Introduction

Quantitative acoustics (ax-QA) is a promising method for the assessment of bone health. Based on the mechanical phenomenon of acoustic wave propagation, ax-QA is completely radiation-free and non-invasive if actuator and sensors are placed onto the skin, as is common practice. Consequently, QA is a prime candidate for widespread monitoring applications and measurements on newborns [1,2] or children [3], for which radiation-based methods such as x-ray imaging, dual x-ray absorptiometry (DXA), or computed tomography (CT) are less well suited. Contrary to radiation-based methods, QA is further inherently sensitive to both the inorganic and organic compounds of bone, and could thus offer complementary information to established methods, particularly for applications such as the detection of osteoporosis [4] or the assessment of bone strength [5,6].

Axial transmission quantitative acoustics (ax-QA) is one form of QA in which an actuator creates mechanical waves inside the bone to be examined and sensors measure the wave propagation along the bone axis [7]. Advantages of ax-QA include the potential to assess a large bone section in a single measurement and the fact that only unilateral access to the bone is required. Typical application sites include the tibia, radius, and ulna, but recently the femur has received increased attention [5,8,9]. While ax-QA has shown promising results for detecting bone pathologies and many material and structural properties [6,7,10–15], the disentanglement of individual effects has emerged as a key challenge for further developments. In a previous work [16], we have therefore proposed a systematic bottom-up approach that investigates the effect of variations in individual bone properties onto the wave propagation, before later progressing to interaction effects. By systematically moving towards a more complex and realistic model, we hope that such an approach will ultimately help unlock ax-QA´s potential to comprehensively assess bone strength.

The thickness of the cortical bone shell is a key determinant of a bone’s mechanical properties—in osteoporosis for example, the decrease in cortical thickness (cTh) is one of the main factors leading to an increase in fragility and fracture risk, high numbers of mortality and morbidity [17,18], and the associated burden on the public health system [19,20]. For these reasons, considerable effort has been made to investigate the measurement of cTh using ax-QA, from numerical simulations and experiments on in-vitro bones [11,21–27] to in-vivo measurements [28,29]. These studies have shown that wave propagation in bone are well described by plate (near 1MHz) and tube theories (near 250kHz), and have yielded valuable insight into the underlying processes, such as the nature of the experimentally observed first-arriving signal (FAS) and energetic late arrival (ELA). Notably, the FAS was identified to correspond to either a S0-like wave mode from plate theory or a lateral head wave [22] and ELA was ascertained to correspond to the fundamental flexural A0-like wave mode. While there are multiple ways to measure cTh using ax-QA, e.g. using higher order modes combined with an inversion technique [30,31], the most common approach is to measure a wave mode at a frequency that shows a strong dependency on thickness. Here, both experiment and theory indicate that the A0-like mode and lower frequencies are more sensitive to cTh than the S0-like mode and higher frequencies [11,32,33]. Despite these findings, the low frequency regime below 250 kHz has remained largely unexplored. While tube theory predicts an increase in the A0-mode’s sensitivity to cTh at lower frequencies, the associated wavelength becomes comparable to the bone’s dimensions at frequencies in the kHz-range. So, even though long bone are well approximated by tubes around 250kHz, at even lower frequencies this approximation could break down. This study therefore investigates whether the sensitivity of the A0-like mode increases in the low-frequency regime as predicted by tube theory or whether the underlying assumptions break down, requiring a more faithful modelling of the bone’s geometry.

Another open question concerns the implications of varying cTh: while plate and tube models have shown suitable in many cases to describe wave propagation in human long bones [24,25,31], these models assume constant thickness. However, the cTh of real bone varies along the bone’s circumference and long axis–sometimes increasing by more than 100% [11]. Here, previous works have investigated the effect of varying cTh on bones and phantoms along the bone’s long axis [11,24,34]. It was found that tube theory can still describe the wave behavior at frequencies of about 250 kHz if the local cortical thickness beneath the measurement site was considered the model’s thickness parameter. Of course, human bone also varies along the long axis, which might make identification of a unique local cortical thickness impossible for ax-QA measurements, in which sensors are spread out across a certain distance along the bone. Because such an ambiguity could have severe consequences for applications that rely on cTh measurements, a better understanding of the effects stemming from varying axial cTh are clearly needed.

The presented study therefore aims to investigate a) the sensitivity of ax-QA measurements to cortical thickness in the kHz-regime, b) the applicability of tube theory in this regime, and c) the influence of varying cTh along the long axis on the bone.

## Methods

### Phantoms

**Fig 1:**
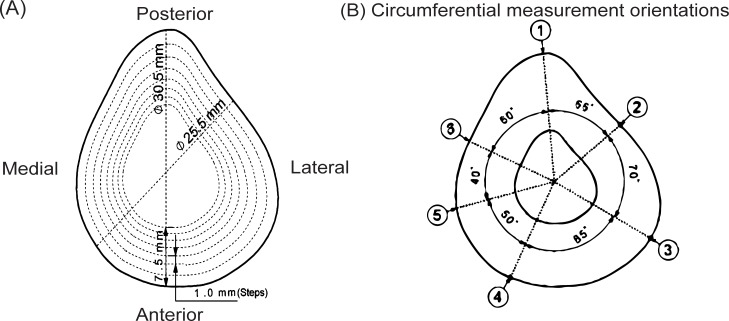
The inner radius of the phantoms is varied to achieve different cortical thickness values; the dotted lines show how the inner surface changes in steps of 1mm to create seven phantoms of varying thickness; b) The six measurement orientations based on angular positions.

**Fig 2:**
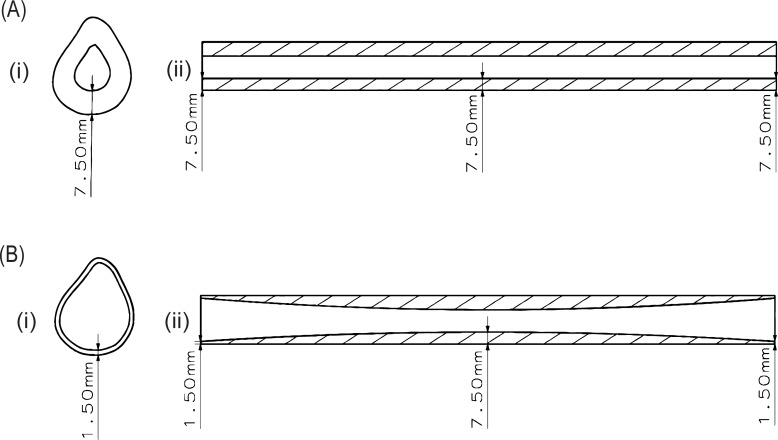
Transversal Cross-section (i) and longitudinal cross-section (ii) of the a) 7.5mm azimuthal phantom and b) the 7.5mm axial phantom. For both types, phantoms with thicknesses between 1.5mm and 7.5mm were manufactured. While the cortical thickness remains constant along the longitudinal axis for the azimuthal phantoms, it varies for the axial phantoms.

For this study, 14 phantoms of length 400mm, with a bone-like cross-section (Fig 1A) and average cTh between 1.5mm and 7.5mm were designed, covering typical in-vivo cTh values of the tibia or femur [35]. In seven of these phantoms the cTh varied around the phantom’s azimuth (“azimuthal phantoms”, Fig 2A) whereas it varied both azimuthally and axially in the remaining seven phantoms (“axial phantoms”, Fig 2B). Because cortical thinning due to ageing or osteoporosis occurs mainly at the endosteal surface [36,37], the outer shape was kept identical for all phantoms while the inner shape was systematically adjusted to achieve varying cTh (Fig 1A). For the axially varying phantoms, the dimensions corresponded to those of the 1.5mm thickness azimuthal phantom at both ends, but the cTh smoothly increased towards the middle (Fig 2B). Six measurement orientations were defined along the circumference of each phantom (Fig 1B) along which the ax-QA measurements were later performed. For each of these orientations, the local cTh at half the phantoms’ length was determined, resulting in 6 local cTh values per phantom (Table 1).

**Table 1:** Cortical thickness values for each phantom and measurement orientation. Local thickness is measured at half the length of each phantom.

Phantom	Measurement orientation	Local thickness (mm)	Average thickness (mm)
Azimuthal/axial 1.5mm	1	1.72	1.5
	2	1.34	
	3	1.58	
	4	1.52	
	5	1.49	
	6	1.41	
Azimuthal/axial 2.5mm	1	2.95	2.5
	2	2.18	
	3	2.66	
	4	2.54	
	5	2.48	
	6	2.32	
Azimuthal /axial 3.5mm	1	4.18	3.5
	2	3.02	
	3	3.74	
	4	3.53	
	5	3.45	
	6	3.23	
Azimuthal /axial 4.5mm	1	5.4	4.5
	2	3.86	
	3	4.82	
	4	4.54	
	5	4.43	
	6	4.14	
Azimuthal /axial 5.5mm	1	6.62	5.5
	2	4.7	
	3	5.9	
	4	5.55	
	5	5.41	
	6	5.05	
Azimuthal /axial 6.5mm	1	7.85	6.5
	2	5.54	
	3	6.99	
	4	6.57	
	5	6.4	
	6	5.96	
Azimuthal /axial 7.5mm	1	8.96	7.5
	2	6.38	
	3	8.06	
	4	7.58	
	5	7.38	
	6	6.87	

The phantoms were manufactured from polyamide 2200 (PA 22) using selective laser sintering. PA22 was chosen because it is 3D-printable, durable and its material properties (density: 0.97 g/cm^3^, elastic modulus: 1.5 GPa, Poisson’s ratio: 0.408, longitudinal bulk wave speed: 1880 m/s, transversal bulk wave speed: 741 m/s) are similar to bone substitute materials used in other studies [31,34,38].

### Measurement setup and protocol

The acoustic measurements in this study were performed using the Bone Stiffness Measurement Device (BSMD), which has been described in detail elsewhere [39]. Briefly, the BSMD consists of a piezo-electric transducer (P-840.20 piezo-stack, PI Ceramic GmbH, Lederhose, Germany) to excite an acoustic wave, multiple acceleration sensors to measure the wave propagation, and a data acquisition system to control the device and to record the measurement data. The acoustic wave was excited by a sine of 3000 Hz, enveloped by a Gaussian with Full-Width-at-Half-Maximum (FWHM) of 2500 Hz. The accelerometers (model 4518, Brüel & Kjael GmbH, Pöcking, Germany) had a sensitivity of 100 mV/G, where G is 9.81 m/s^2^, and data was sampled at a frequency of 96kHz.

This experiment utilized a custom-made mounting platform to achieve high reproducibility in transducer, sensor and specimen placement. The phantom was supported two U-clamps at both ends, with foam providing acoustic decoupling between phantom and mounting platform.

**Fig 3:**
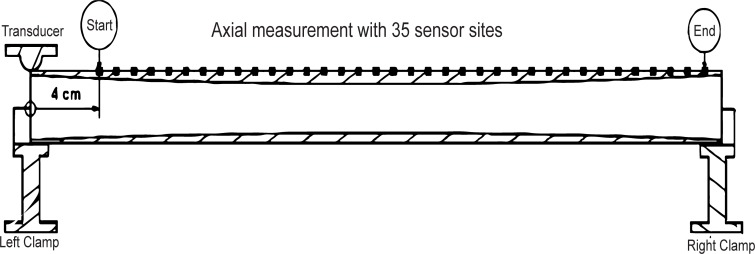
Illustration of the measurement setup and the 35 sensor positions involved in one ax-QA measurement.

The transducer was mounted, facing radially inwards, 0.5 cm from the end of the phantom, which also acted as the origin of the coordinate system (Fig 3). One acceleration sensor was placed in-line with the transducer at a distance of 4cm. In this configuration, a wave was induced and 2000 data points were measured, corresponding to approximately 20ms. The sensor was then repositioned 10mm along the phantom’s axis and another measurement was performed; this process was repeated until the sensor reached the end of the phantom. Overall, this procedure resulted in a collection of 35*2000 space-time data for each of the 14 phantoms and for each of the 6 orientations (Fig 1B).

### Signal analysis

To extract the phase velocity, each raw measurement was first windowed using a moving hamming window to remove the reflections occurring at the ends of the phantom while preserving the first transit of the wave pulse. The window width was 400 samples, corresponding to about 4ms, and its centre was placed at 11 ms for the first sensor position (at 4cm). The window was moved with a speed of 480 m/s between sensors, corresponding to a centre position of about 11,7 ms at the last sensor position. Changes to window width or window speed (e.g. 200 m/s instead of 480 m/s) did not meaningfully alter our results. Plots of the raw data, the data windowed with 480m/s, and the data windowed with 200 m/s, as well as their corresponding spectra is presented in the electronic supplementary material.

**Fig 4:**
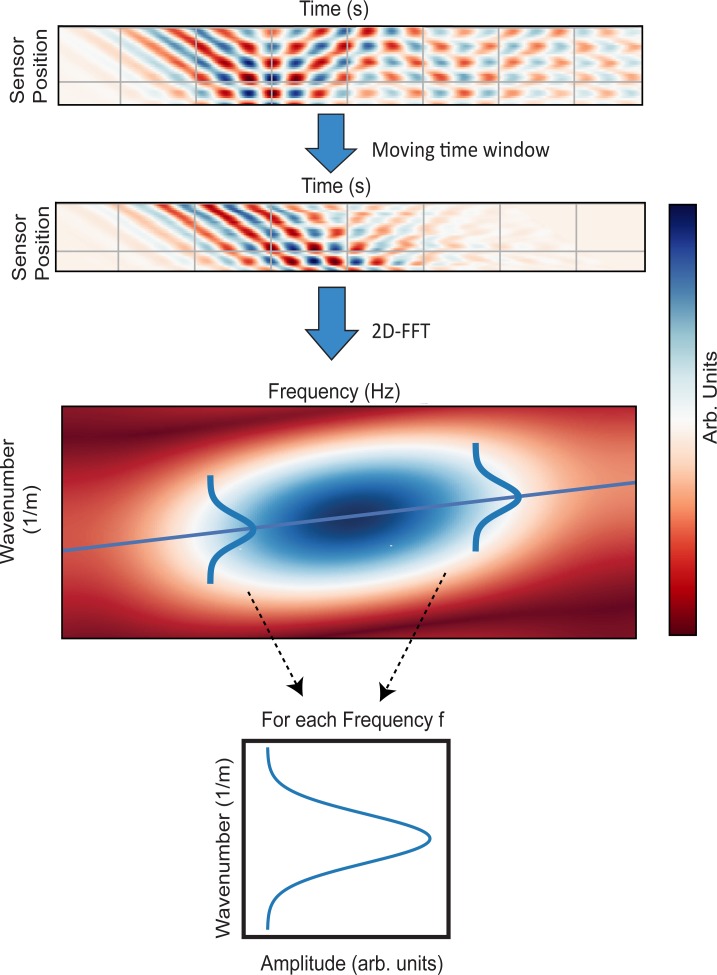
Illustration of the analysis procedure consisting of a 2D Fast-Fourier-Transform, followed by fitting of a linear Gaussian model, resulting in an estimate for the mode’s wavenumber as a function of frequency.

The filtered data was then transformed to frequency-wavenumber space using a 2D Fast-Fourier-Transform (2DFFT) with 25000 and 3500 points, which corresponds to about 10 times the acquired sample number and 100 times the sensor number (Fig 4).As the phase velocity is given by *c* = *f*/*v*, the phase velocity *c* can be found by extracting the linear wavenumber *v* for each frequency *f* from this transformed data. Towards this end, we fit a linear Gaussian model of the form

$$A(f)\;N(x=\nu |\mu =\alpha \;f+\beta ;\Omega^{2}=\sigma^{2})$$ (Eq 1)

to the data using non-linear least squares. The Gaussian form was chosen as a close approximation of the sinc-like behavior, which is predicted theoretically because the 35cm of total sensor array length can be considered as a rectangle function, the Fourier transform of which is the sinc function. Here *N*(*x*|*μ*;Ω^2^) denotes the unit Gaussian of variable *x* with mean *μ* and variance Ω^2^, and *A*(*f*) is the amplitude dependent on frequency f with assumed Gaussian shape *A*(*f*) = *A*_0_ *N*(*f*|*ζ*; *η*^2^). As a result, the free parameters to be optimized in the fitting procedure were *A*_0_,*ζ*,*η*,*σ*. The linear dependency of the fitting function on frequency is motivated by the fact that the dispersion relation in the narrow frequency range of ~2500Hz-3500Hz is expected to be approximately linear [40]. Optimization across multiple frequencies at the same time is less prone to experimental noise and side peak effects and thus more stable than fitting each frequency separately [16].

After fitting, the phase velocity and its uncertainty were calculated from $c(f)=\frac{f}{\nu }=\frac{f}{\alpha f+\beta }$ and $\Delta c=f\frac{\Delta \nu }{\nu^{2}}=f\sqrt{{\Delta \alpha }^{2}f^{2}+{\Delta \beta }^{2}}/\nu^{2}$, which follows from Gaussian error propagation [41]. In the following, we present our results at the experimental excitation frequency of *f* = 3000*Hz*.

As a side note, this procedure was repeated using five other fitting functions, differing slightly in the functional form of the amplitude *A*(*f*) and the Gaussian’s variance *σ*(*f*). The phase velocities resulting from the six fitting functions showed pairwise correlations of about 0.8–0.9 and we therefore only present results for the fitting function Eq 1. Details on all investigated fitting functions (S1 File) and the correlation matrix for their results (S1 Fig) can be found in the supplementary material, and phase velocity results for the remaining five fitting functions can be found in the electronic supplementary material.

## Results and discussion

**Fig 5:**
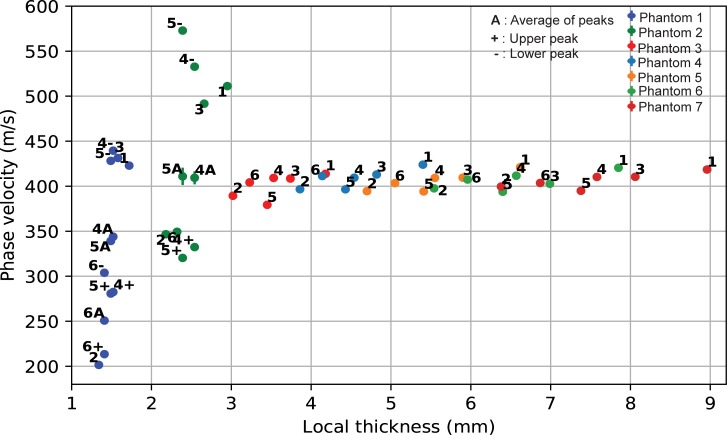
Phase velocity as a function of local thickness for each azimuthal phantom and measurement orientation (1–6).

**Fig 6:**
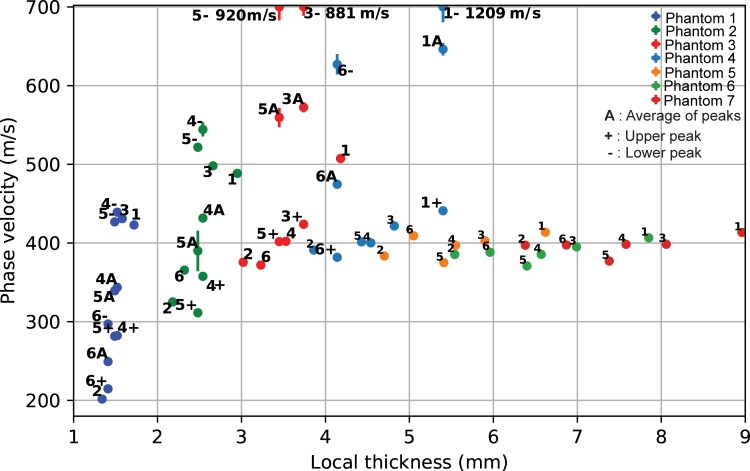
Phase velocity as a function of local thickness for each axial phantom and measurement orientation (1–6).

This study investigated the effect of changing cortical thickness on low-frequency ax-QA measurements. Towards this end, 14 homogenous bone phantoms with a femur-like cross-section and a) axially varying cortical thickness or b) axially and azimuthally varying cortical thickness were measured using ax-QA. The measured phase velocities for both phantom types depended on local cTh up to 3-4mm thickness (Fig 5 and Fig 6). For higher cTh, the phase velocity remained constant at 400 m/s, which is about half the transverse wave speed of the bone substitute material. Notably, the results from both types of phantoms were nearly equal (Pearson correlation coefficient: 0.94), indicating that the cTh thinning towards the ends only weakly affected wave propagation when compared to the phantoms with axially constant cTh.

**Fig 7:**
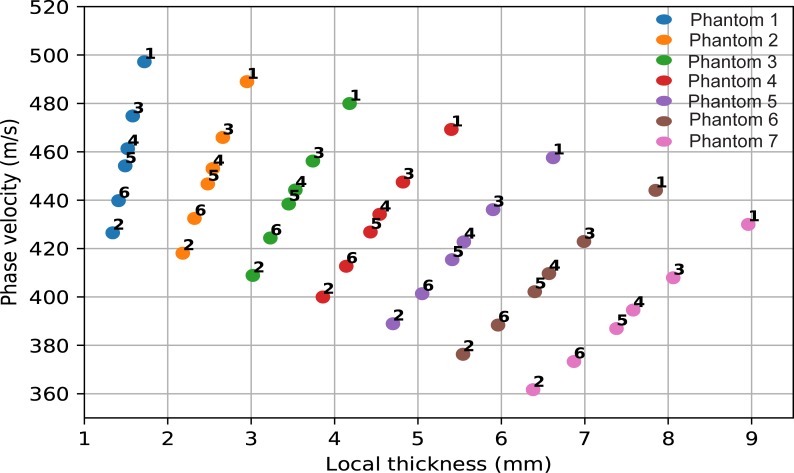
Phase velocities predicted by exact elastic tube theory for thickness values corresponding to the local cTh of each axial phantom and measurement orientation (1–6).

Exact elastic tube theory fails to predict the measured data, while giving the right order of magnitude for higher cortical thickness–while theory predicts a decrease in phase velocity with increasing cortical thickness (Fig 7), a more complex but generally increasing behavior was observed experimentally (Figs 5 and 6). This disagreement could indicate that the complex geometry of the phantoms cannot be approximated by a tube at the low-frequencies used in our study. Even though long bones, such as the tibia, can be well described by tube or even plate theories at higher frequencies [24,25,31], the wave behavior depends critically on the ratio between the wavelength *λ* and the characteristic length scales of the system such as cortical thickness *d* or radius of curvature *r*. While half-space theories have been successful when *λ*≪*d*,*r*, plate or tube theories have been required when *λ*~*d*. It thus seems plausible that for even lower frequencies and longer wavelengths, when *λ*≫*d*,*r*, the overall shape might become a key factor, rendering theories based on simple geometries insufficient. Because the longitudinal and transversal wavespeeds of PA2200 are about half the values found in cortical bone [42], this effect could arise earlier for the longer wavelengths expected in bone. While such a behavior would explain the observed mismatch between tube theory and our experiment (for which the wavelength is in the cm range), it remains to be confirmed by future numerical and experimental studies investigating the influence of shape over a wide range of frequencies.

We would like to note here, that our theoretical results are in agreement with the solutions of tube theories with regards to cTh presented in Fig 1 of another study [34]. Here, their increasing phase velocities as function of cTh appears to mismatch our decreasing velocities. However, this discrepancy is resolved by noting that the ratio of outer to inner radius *a*/*b* is assumed to be constant for their results. For our study (and the experimental part of their study) the increase in cTh leads to a change in the *a*/*b* ratio, meaning that measurements from a single phantom come from different *a*/*b* curves. Depending on the range of $\frac{h}{\lambda_{l}}=h\frac{f}{c_{l}}$ values, where h is the local thickness, f the frequency, and *λ*_*l*_,*c*_*l*_ the longitudinal bulk wavelength and wavespeed, this “jumping” between *a*/*b* curves has different consequences: for the low $\frac{h}{\lambda_{l}}$ values between 0.003 and 0.006, an overall decrease in phase velocity with increasing cTH is predicted, while at $\frac{h}{\lambda_{l}}$ values higher than about 0.4 a smooth increase is predicted, which has also been experimentally observed [24,32,34].

Beside the general increase and plateauing with increasing cTh, our results showed additional variation dependent on phantom measurement orientation. This possibly indicates that local cortical thickness is an insufficient measure, and that other local properties are required to fully describe wave propagation in such shapes. However, additional analyses of the phase velocity results as a function of local ratios between outer and inner radius *a*/*b* and local radius of curvature did not yield any apparent functional relationship. While it was found previously that local cTh might not be uniquely defined for measurement orientations near points with high curvature [24,32], none of our measurement orientations seems to correspond to such a situation. Furthermore, the inconsistencies of relative phase velocity differences between measurement orientations at lower cTh makes it unlikely that a redefinition of local cTh would be sufficient to explain our results.

Other studies on bone phantoms [32,34] and human radii [11,34] have ascertained that measurement of the A0-mode at about 200kHz can measure cTh between 2-12mm. Alternatively, a recent multimodal approach using frequencies between 200kHz and 1MHz has shown promising in-vivo results [31], for the first time combining information from multiple modes and frequencies towards the determination of cTh. While our results indicate that measurement of the A0 mode at frequencies as low as 3kHz could potentially provide high sensitivity for cTh lower than 4mm, the underlying phenomena and the influence of overall bone shape prohibit direct translation. Until these processes are better understood, other approaches remain preferable for the determination of cTh.

Even though the measurements in both types of phantoms agreed well, the phase velocity measurements in the low cortical thickness are complicated by the appearance of double peak structures in the 2D-FFT, which make unambiguous determination of phase velocity difficult. These double-peaks appeared only for certain measurement orientations and phantoms, but remained when the experiment was repeated. As most ax-QA approaches include a 2D-FFT in one form or the other, further research into the reasons for these double peak appearances might be warranted, especially for wide-frequency inversion approaches based on multiple modes [31]. When properly accounting for such effects, our results reassuringly indicate that axial cTh variations that would typically arise in a clinical setting should not pose a fundamental obstacle to 2D-FFT analysis.

Clearly, our phantoms can only be considered a rough approximation of a real bone. The use of a homogenous, isotropic substitute material and a simplified bone structure limit the transferability of our results to in-vivo measurements. While these simplifications were made purposefully to follow the systematic approach of investigating effects caused by individual properties, we expect the observed effects of changing cortical thickness to hold for real bone—even though they could possibly be overshadowed by other effects. For example, pores and bone marrow have substantially different acoustic properties compared to air and are known to modify wave propagation [15,24,27,43]. These effects, as well as the effects arising from anisotropy [44,45] or a more realistic shape [15,46], should be investigated in future work. Even though the smooth and gradual axial variations in cTh did not strongly alter wave propagation, more abrupt changes could cause other effects, such as axial reflections from the thickness discontinuity. Because our study only uses a narrow frequency range around 3kHz, the transition process from the regime in which tube theory holds (about 250kHz) to the regime where it fails remains unknown. Possibly, there exists a frequency range for which the sensitivity to cTh is higher than at 250kHz but the shape can still be approximated by a tube. Overlying soft-tissue complicates in-vivo application of ax-QA. Here, compensation methods have shown promising results, even for challenging locations such as the femur [8,47,48].

In conclusion, low-frequency ax-QA exhibited a high sensitivity to local cTh lower than 4mm, albeit with a complex, not yet understood pattern. Tube theory was unable to describe the wave’s behavior in the kHz range, indicating that due to the arising long wavelengths the bone can no longer be approximated by a tube, thus requiring careful consideration of the bone geometry’s effects. Lastly, our results indicate that slowly varying cTh along the bone’s long axis did not strongly affect wave propagation as measured by ax-QA measurements.

## Supporting information

S1 Fig Analysis function correlation matrix. Pearson correlation coefficients between the phase velocity results of the 6 investigated fitting functions (a-f).
(EPS)

S1 File Fitting function details. Details on the different fitting functions used to calculate the phase velocity.
(DOCX)
