# Supplementary material for: Sensitivity of low-frequency axial transmission acoustics to axially and azimuthally varying cortical thickness: A phantom-based study
Source: PLoS One. 2019 Jul 17;14(7):e0219360. doi: 10.1371/journal.pone.0219360 (PMC6636721; doi:10.1371/journal.pone.0219360)
Supplement: S1 File — Details on the different fitting functions used to calculate the phase velocity. (DOCX) [file pone.0219360.s002.docx]

S1 File: Details on the different fitting functions used to calculate the phase velocity.

a) ampConst_sigmaFree, for which $A\left( f \right)=A$ independent of frequency, and $\sigma$ was a free parameter;

b) ampConst_sigmaFixed, for which $A\left( f \right)=A$, $\sigma$ is set to $\sigma=2.8571$, which is the FFT limited resolution arising from the total measurement length of 35cm.

c) ampGaussian_sigmaFree, for which the amplitude was assumed to have a Gaussian shape $A\left( f \right)=A_{0} N(f|\zeta; \eta^{2})$, with $A_{0}, \zeta, \eta, \sigma$ being free parameters.

d) ampGaussian_sigmaFixed, for which the amplitude $A\left( f \right)$had the same functional form as in c), and $\sigma=2.8571$ as in b)

e) ampsemigaussian_sigmaFree; for which $A\left( f \right)=A_{0} N(f|\zeta; \eta^{2})$ and $\zeta=3000 Hz, \eta=1000Hz$were set to the experimental excitation frequency and bandwidth, and $\sigma$ was a free parameter

f) ampsemigaussian_sigmaFixed, for which the amplitude $A\left( f \right)$had the same functional form as in e), and $\sigma=2.8571$ as in b)
